# Factors Affecting Psychosocial Distress in Adolescents and Young Adults with Cancer: BRIGHTLIGHT Cross-Sectional and Longitudinal Cohort Study Results

**DOI:** 10.3390/cancers17071196

**Published:** 2025-03-31

**Authors:** Chana Korenblum, Rachel M. Taylor, Lorna A. Fern, Rachael Hough, Bethany Wickramasinghe

**Affiliations:** 1 The Hospital for Sick Children, Princess Margaret Cancer Centre, Temerty Faculty of Medicine, University of Toronto, Toronto, ON M5G1X8, Canada; 2 Centre for Nurse, Midwife and Allied Health Profession Led Research (CNMAR), University College London Hospitals NHS Foundation Trust, Department of Targeted Intervention, University College London, London NW1 2PG, UK; rtaylor13@nhs.net; 3 Cancer Clinical Trials Unit, University College London Hospitals NHS Foundation Trust, London NW1 2PG, UK; lorna.fern@nhs.net; 4 Children and Young Peoples Cancer Service, University College London Hospitals NHS Foundation Trust, Cancer Institute, University College London, London WC1E 6AG, UK; rachaelhough@nhs.net; 5 Department of Targeted Intervention, University College London, London NW1 2PG, UK; bethany.wickramasinghe.19@alumni.ucl.ac.uk

**Keywords:** cancer, young people, adolescents, young adult, BRIGHTLIGHT, mental health, distress, depression, anxiety

## Abstract

Background/Objectives: AYAs face unique psychosocial challenges compared to younger children and older adults. Little published data exists about symptoms of psychological distress in AYAs, particularly in large cohorts over time. This study aimed to (1) explore relationships between distress and variables known to impact mental health, (2) examine distress over time, and (3) consult with a patient and public involvement group (Young Advisory Panel, YAP) to contextualize the results. Methods: Secondary data analyses were conducted from a multicentre longitudinal cohort study, which recruited 830 participants aged 13–24 years newly diagnosed with different cancers. The mean age was 20.1 (SD 3.3) and 45% were female. Bespoke surveys, including a validated measure of psychosocial distress, were administered five times over 3 years following diagnosis. Participatory workshops were conducted with the YAP to gather their impressions of the study results and inform conclusions. Results: At the first time point, higher social support was associated with higher distress scores (β = 0.202, *p* < 0.001) and AYAs with higher depressive symptoms were more likely to see a mental health professional (*p* < 0.01). Over time, anxiety symptoms remained stable (*p* = 0.090); however, depressive symptoms improved (*p* < 0.001). The YAP highlighted the mixed effects of social support and the under-recognition and undertreatment of distress. Conclusions: This information identifies key targets, such as social support, that can inform the design of personalized screening and intervention tools. It also highlights specific timepoints at which these efforts can be directed to mitigate distress and improve quality of life. Future research should include longer-term follow-up, qualitative explorations of distress along the disease trajectory, and ongoing co-production efforts.

## Simple Summary

Adolescents and young adults with cancer (AYAs) have biopsychosocial needs that differ from younger children and older adults. Facing cancer during this complex developmental stage can result in mental health consequences, historically not adequately addressed by pediatric or adult healthcare systems, nor by researchers. This study adds a deeper understanding of risk and protective factors for distress in AYAs, including the paradoxical impacts of social support. It also offers a longitudinal description of symptoms, with depression improving over time while anxiety remained stable. Future implications include informing the design of developmentally tailored screening and intervention tools to mitigate distress in this understudied population.

## 1. Introduction

Each year worldwide, there are approximately 1.2 million new cases of cancer in young people aged 15–39 years, representing 6% of all new cancer diagnoses [1]. After accidents, cancer ranks as the next most common cause of death in young people in the United Kingdom (UK) [2]. Adolescents and young adults with cancer (AYAs) are defined in the UK as those aged 16–24 [3] and new diagnoses number approximately 2400 annually [4]. Cancer in AYAs presents distinct biological and psychosocial considerations that require special attention [5,6].

Cancer biology, genomics, and host pharmacokinetics in AYAs differ from younger children and older adults and are poorly understood [7]. Among the most common cancer types occurring in this age group are lymphomas, thyroid carcinomas, testicular tumours, and melanomas [5,8]. Prolonged and complex pathways to diagnosis are common, due in part to a lack of awareness in the medical community and general public, risks of treatment side effects are higher, and survival rates have not improved the way they have in other age groups [9,10,11,12]. Additionally, enrolment in clinical trials is very limited, preventing advances in new drug development, and thus impacting cure rates [8,13,14]. Long-term morbidities from treatment include chronic cardiac, musculoskeletal, neurocognitive, and endocrine conditions, as well as the risk of second primary cancers [15,16]. In those who survive into older age, their life trajectories may be profoundly altered, representing a significant number of disability-adjusted life years impacting individuals, families, and society [17,18].

Although cancer is disruptive at any age, a diagnosis during this phase of life has a particularly acute psychosocial impact, affecting body image, peer and romantic relationships, fertility, family dynamics, education, employment, future life plans, and mental health [19,20,21]. Adolescence and young adulthood are inherently dynamic periods of development without a cancer diagnosis, and these life stages have become increasingly complex in recent times [22]. Nearly three-quarters of adult mental health problems are thought to start during this pivotal life stage [23]. Cancer fundamentally disturbs this already precarious developmental trajectory, isolating youth precisely when they want to fit in [24]. Furthermore, AYAs have a greater ability compared to younger children to understand the severity of their situation but have fewer emotional, informational, and practical resources as compared with older adults to help cope [25].

Not only do AYAs with cancer have unique biopsychosocial needs, but they fall into the gap between cancer care services designed for younger children, where the average age is 6, and older adults, where the average age is 66 [26,27]. They are “squeezed between, and insufficiently addressed by, the achievements of the pediatric oncology world on the one hand and the weight of cancer burden on adult cancer services on the other” [28]. Described as ‘no man’s land’ or ‘the lost tribe,’ AYAs experience a lack of autonomy, peer connection, support to complete developmental milestones, appropriately trained staff, and tailored healthcare environments [29,30]. The advent of high-quality AYAs specialist services and support organizations in many countries has begun to address AYAs needs, but these services currently only cover a minority of AYAs worldwide and generally require more comprehensive resources to fully support this population. Therefore, it is unsurprising that AYAs with cancer experience high levels of psychological distress [24]. Psychological distress, defined for this paper as an overall measure of anxiety and depression [31], can impact adherence to treatment, overall morbidity and mortality, experience of healthcare services, as well as quality of life [32]. However, evidence about anxiety and depression in AYAs with cancer is limited, including prevalence in large samples and symptom trajectories over time. Understanding the distinct psychological needs of this population may help reveal mechanisms for addressing these disparities and mitigate the negative consequences of cancer care.

The body of evidence examining mental health symptoms in AYAs with cancer is limited but has gradually increased over the last two decades. This growth may be attributed to a rising recognition of the importance of providing supportive care for cancer patients as survival rates improve, in parallel to searching for new cures [33]. Another contributing factor may be the change in Western societal attitudes toward an awareness of mental health issues [34].

However, there are methodological challenges in AYAs cancer research including varying definitions of the AYAs age range, studies that group AYAs in with other age categories, small sample sizes, multiple cancer types, lack of validated measures for this age group, and few longitudinal studies [33,35].

Estimates of the prevalence of distress in AYAs with cancer are based on a small number of systematic reviews and descriptive studies, with age ranges including 12–25, 15–24, and 15–39, among others [20,36,37]. Rates of anxiety range from 8 to 55% and depression from 13 to 47% [31,38,39,40]. Compared to older people with cancer, siblings, and peers, AYAs face higher risks for these conditions [31,41,42]. Some evidence suggests that AYAs experience improvements in anxiety and depression over time, though the trajectory and timing of these changes vary across studies [43]. The generalisability of much of the research on this issue is questionable, due to small sample sizes, inconsistently applied psychometric measures, variation in diagnoses and complexity of treatment across studies, and primarily cross-sectional designs. However, despite these limitations, there is consensus and concern that psychological needs are high and remain unmet, and that an understanding of the developmental context of this population is crucial and lacking [35,37]. Contextual factors such as patient, social, institutional, and healthcare system level resources are also important considerations [20].

Multiple risk factors have been identified in the literature as contributing to increased distress in AYAs with cancer [20,44,45]. Female gender [46,47,48], older age at diagnosis [47], migration background [46], and having a brain tumour diagnosis [40] have all been implicated. However, factors that feature consistently across multiple studies include various proxies of poor social support (e.g., difficulties with social relationships, not being in a partnership, social isolation, and poor family functioning) as well as disease-related features including illness relapse or a higher number of late effects [20,40,45,49]. Certain psychological characteristics have also been identified, such as maladaptive coping skills, low self-image, and identity issues [40,48]. These commonly arising factors are explored in more detail as follows.

Social support is defined by the National Cancer Institute’s Dictionary of Cancer Terms [50] as “a network of family, friends, neighbours, and community members that is available in times of need to give psychological, physical, and financial help”. Social support has been observed to alleviate distress in AYAs and potentially improve with intervention [51]. A defining developmental task of this life stage is to individuate from family and shift toward peer influences, a challenge even when healthy [52]. A cancer diagnosis and its treatment can disrupt a young person’s evolving social network to varying extents depending on the illness severity, treatment intensity necessitating repeated or prolonged hospital admissions, emotional development of the patient, and capacity of family and friends to cope with the situation [20,51]. Qualitative focus groups with 25 AYAs aged 16–39 at the time of the study identified social isolation as a major psychosocial dilemma, encompassing separation from peers due to treatment, loss of independence due to reliance on caregivers, and feeling like an outsider in the medical setting (either the youngest person in the adult clinic, or the oldest person in the pediatric clinic) [45]. Limited evidence from longitudinal observational studies proposes that social functioning in AYAs at diagnosis is low, and though it improves over time, it remains reduced compared to healthy peers [53]. While the general trend across the literature shows increasing distress with worsening social support, this area remains understudied.

Disease-related features in AYAs associated with higher levels of distress include diagnoses such as brain tumours, head and neck cancers, and testicular cancers [40,54]. In a population-based study of 572,000 AYAs, rates of suicide—a serious potential consequence of extreme distress—were higher among those with metastatic illness [54]. This corroborates evidence that relapsed disease predicts poorer emotional health [55]. Long-term treatment complications detailed above also contribute to worsening mental health [46,56]. Following from the evidence, specific features of an AYA’s illness, while not necessarily modifiable, could inform the early stratification of patients to individualized support.

Finally, psychological characteristics have been correlated with increased distress in AYAs [57]. In a systematic review of predictors of psychiatric disorders in AYAs, four studies found that AYAs engaged in maladaptive coping strategies, such as wishful thinking and avoidance, are more often compared to healthy peers [40]. Self-image and identity issues in a cross-sectional, multi-centre study of 196 AYAs were associated with elevated distress [48]. However, few if any studies have directly examined the relationship between pre-existing mental health conditions and distress. These conditions could be screened for at diagnosis, helping allocate support to those who may need it most.

Little is known about the change in symptoms of distress in AYAs over time along the disease trajectory. An older prospective observational study of 61 Swedish adolescents aged 13–19 indicated improved psychosocial functioning over the first 18 months after diagnosis [44]. A follow-up study of the same cohort over a much longer period of 10 years showed a non-linear pattern, with anxiety and depression declining around the 2-year mark, and then increasing gradually over the subsequent 8 years [58]. Another similarly designed study of 514 German AYAs aged 18–39 found rates of anxiety and depression remained stable over two time points (the first within 4 years of diagnosis, and the second 12 months later) [49]. There have been few attempts worldwide to quantify this problem longitudinally. Qualitative exploration is also needed, to examine possible reasons behind observed time course patterns.

The objectives of this study were to illustrate the mental health burden in a large, geographically diverse, well-characterized cohort of AYAs with cancer aged 13–24 at diagnosis and examine risk and protective factors that affect mental health in the cohort. The primary aim was to explore relationships between distress and variables described in the literature known to impact mental health, including social support, disease severity, pre-existing mental health conditions, and contact with mental health professionals. The associated hypotheses were that lower levels of social support, higher disease severity, presence of a pre-existing mental health condition, and contact with mental health professionals would be associated with higher levels of distress. The secondary aim was to examine these associations over time. Distress was hypothesized to decrease with passing time. The final aim was to consult with an established patient group of young people about the results and seek their interpretation based on their own experiences.

A deeper understanding of the distinct psychological needs of this population will help inform the design and evaluation of individualized, effective screening processes and interventions, targeted at key time points, to reduce distress and mitigate the negative biopsychosocial consequences of cancer care during this dynamic life stage.

## 2. Materials and Methods

### 2.1. Study Design

To characterize distress in AYAs and examine changes over time from diagnosis, a secondary analysis of raw data from the BRIGHTLIGHT study was conducted. BRIGHTLIGHT was a national programme of research, central to which was a multicentre, prospective, longitudinal cohort study, aiming to determine whether specialist care for AYAs in England was associated with improved outcomes [59,60,61]. A patient and public involvement group (PPI) Young Advisory Panel (YAP) of AYAs with cancer were involved in BRIGHTLIGHT from the outset as co-researchers, naming the study, developing the design, research questions, and outcome measures, disseminating results, and identifying future areas for research. PPI in this context is understood as conducting research together with the public instead of on them [62].

### 2.2. Participants and Setting

The BRIGHTLIGHT cohort consisted of 1114 young people aged 13–24 years, recruited within 4 months of a new cancer diagnosis, between 2012 and 2015 from 97 hospitals across England. Recruitment primarily occurred within adult cancer services, but in hospitals offering both child and adult care, individuals aged 13–16 were also enrolled. The cohort represented one-fifth of the total population diagnosed at the time in the UK. Those who did not participate in the cohort were similar in age and ethnicity to cohort participants but had a lower proportion of males and different proportions of cancer diagnoses (lower proportions of leukemia and lymphoma, germ cell tumours and bone tumours and higher proportions of brain tumours, skin cancers, and carcinomas compared to the BRIGHLIGHT cohort).

People with language or sensory impairments impacting communication were included. Young people were excluded if they had a prognosis of less than 6 months from the time of diagnosis, received a custodial sentence, or were unable to complete a survey [60]. The Confidentiality Advisory Group of the Health Research Authority (ECC 8-05[d]/2011) and the London Bloomsbury NHS Research Ethics Committee (reference 11/LO/1718) approved the study, including activity involving the YAP [62].

### 2.3. Procedures and Measures

Data were collected through a study-specific survey, administered by an independent research organization across five time points: 6, 12, 18, 24, and 36 months following diagnosis [60]. Interviews were conducted in person at the first time point and by telephone or online for the subsequent time points. The co-designed survey (available under licence from https://xip.uclb.com/i/healthcare_tools/brightlight_wave1.html, accessed on 30 January 2025) contains demographic items, validated outcome measures, and questions about cancer care.

This paper reports socio-demographic data and clinical characteristics shown in the literature to impact mental health. Information was collected about participants’ age, gender, ethnicity, marital status, geographic location, socioeconomic status, and employment status. Clinical characteristics included cancer type, time to diagnosis, disease severity at diagnosis, prognostic score, and treatment type. The severity of illness was determined using a bespoke grading system incorporating the burden of symptoms, treatment, and late effects, as well as predicted survival. A grade of low, intermediate, or high severity was assigned to each cancer type [60]. An existing scoring system was used to determine patient prognosis at the first time point, using anticipated 5-year survival to separate young people into those with an expected survival of less than 50%, 50–80%, and above 80% [63,64].

This paper also details the psychosocial characteristics of the cohort to approximate their mental health status, including scores on the Hospital Anxiety and Depression Scale (HADS) [65], the Multidimensional Scale of Perceived Social Support (MSPSS) [66], and the self-reported presence or absence of a pre-existing mental health condition.

The HADS was used to measure patient-reported distress, including symptoms of anxiety and depression. Summary scores range from 0 to 21, with 8–10 representing borderline and 11 or higher representing moderate to severe levels of anxiety and depression [67]. The HADS is suitable for individuals with cancer because it does not include potentially confounding questions about physical symptoms [68]. It has been found to be reliable and validated for use in the cancer population [69,70].

Social support was measured using the MSPSS, which identifies an individual’s perceived level of social support from family, friends, and significant others [66]. Example items include “I get the emotional help and support I need from my family”, “I have friends with whom I can share my joys and sorrows”, and “I have a special person who is a real source of comfort to me”. The total support score is reported as an average ranging from 1 to 7. Low support is indicated by a total score of 1–2.9, moderate support by a score of 3–5, and high support by a score of 5.1–7. The measure also has domain scores for support from family, friends, and significant others. The MSPSS has been validated and shown to be reliable in people with cancer [71,72].

Data on whether young people were offered and had contact with a mental health professional at the first time point were also collected, to assess referral numbers and service utilization.

Covariates were selected a priori based on established literature linking psychosocial distress in AYA cancer populations to demographic and clinical factors.

### 2.4. Data Analysis

Descriptive data for demographics were collected, including means and standard deviations, medians and interquartile ranges, frequencies, and percentages. Statistical analyses were performed using IBM SPSS Statistics version 29.0.2.0 software for Mac. A value of *p* < 0.05 was taken as statistically significant with 95% confidence intervals calculated.

Poisson regression was used to examine the association between distress and social support, adjusting for confounders including gender, age, marital status, socioeconomic status, employment status, and ethnicity. This type of analysis was chosen over linear regression as the former summarizes the outcome as an average rate, which is more appropriate for discrete variables, whereas the latter summarizes the outcome as an average.

One-way ANOVAs were performed to elicit the relationship between distress and disease severity, with Bonferroni corrections for post hoc analyses to determine where significant differences lay. Independent samples *t*-tests were used to explore distress and pre-existing mental health conditions, as well as contact with mental health professionals. Mann–Whitney U tests were used for small sample sizes. A repeated measures ANOVA was used to analyze the change in distress across the five time points.

### 2.5. Patient and Public Involvement

To help inform the interpretation and enhance the validity of the study results, all ten active members of the BRIGHTLIGHT YAP were invited to contextualize the findings through their own cancer experiences. Two participatory workshops were conducted online with the YAP and co-facilitated by the research team. The goals of the workshops were to present the key study findings to the YAP, examine their impressions of the results, and explore insights for future research and clinical directions. All participants consented to participation, recording, and using anonymized feedback. Before each workshop, the YAP were given agendas, consent forms, and a written summary of the study results.

The workshops were 1.5 h each and involved a slide presentation of key study findings, followed by a Zoom (version number 6.3.11 (50104)) interactive whiteboard exercise and a focus group discussion [73]. For the first workshop whiteboard exercise, four headings were posted across the top of the board representing each of the variables to be discussed (social support, gender, disease severity, and pre-existing mental health conditions). Three questions were posted down the left side of the board to stimulate thought and facilitate participation for those with treatment-related cognitive difficulties (‘Does this make sense and why?’, ‘How does this relate to your experience?’, and ‘Any other thoughts?’).

In the second workshop, the five time intervals assessed were posted across the top of the board and ‘things I was anxious about’ and ‘things I was worried about’ were posted down the left side. During the whiteboard exercise in both workshops, time was allocated for YAP members to select a ‘virtual sticky note’ upon which to type their thoughts about the results, and ‘stick’ on the whiteboard under the relevant heading. The YAP were sent copies of the whiteboards beforehand, in case they wanted to prepare their thoughts in advance.

During the focus group discussion which followed the whiteboard exercise, members of the YAP were asked for any additional interpretations and retrospective views on how the results may or may not have resonated with their own experiences. A topic guide was used to navigate the conversation. During and after the workshops, the BRIGHTLIGHT team monitored for any distress and followed up with a mental health check-in. Participants were remunerated for their involvement, as per the National Institute of Health and Care Research PPI guidelines [74].

The workshops were held on Zoom under a secure subscription account and were digitally recorded. The YAP selected Zoom as their preferred platform due to the user-friendly whiteboard function. Transcripts were produced using Otter AI software (version number 3.52.240617). The content of the whiteboards and workshop transcripts were reviewed independently by three members of the research team, highlighting key themes through the categorization of responses. The themes were then reviewed and summarized by the research team during a group discussion. A summary report on the workshop was prepared and given to the YAP.

## 3. Results

Demographic, clinical, and psychosocial characteristics will be presented first. This will be followed by the quantitative findings, each of which will then be contextualized with interpretations from the YAP workshops.

### 3.1. Demographic, Clinical, and Psychosocial Characteristics

Eight hundred and thirty AYAs completed the survey at the first time point. Sociodemographic characteristics are shown in Table 1. The mean age was 20.1 (SD 3.3), 45% were female, the majority were white and 73% identified as single/divorced. Most were working or in school (64%) and socioeconomic status was distributed approximately evenly across five indices of multiple deprivations. Approximately 50% of the cohort-self identified as being on treatment.

The clinical characteristics of young people are outlined in Table 2. The most common cancer types were lymphoma, germ cell tumours, and leukemia, and most had a prognosis >80%.

In terms of psychosocial characteristics (Table 3), mean distress scores were below borderline for anxiety and depression, indicating low levels of distress on average at 6 months after diagnosis. Total social support scores were also low, with a median of 1.5 (IQR 1.1–2.2).

Very few young people (n = 7, 0.8%) identified as having a pre-existing mental health condition. Most (n = 628, 74.8%) were offered support from a psychologist, counsellor, psychotherapist, social worker, youth worker, or activity coordinator, and 534 (64.3%) met at least one of these professionals for at least one session.

### 3.2. Quantitative Findings

There was a significant relationship between social support and anxiety levels (β = 0.202, *p* < 0.001), with higher total social support scores associated with higher anxiety scores (Table 4). The relationship was significant for depression in the same direction (β = 0.227, *p* < 0.001), with higher social support scores also associated with higher depression scores (Table 4). Examining the domain scores of social support revealed the same relationships.

When adjusting for confounders, the female gender was associated with significantly higher anxiety (β = 0.696, *p* < 0.001) and depression (β = 0.729, *p* < 0.001), while older age was associated with significantly higher anxiety (β = 1.022, *p* = 0.002) but not depression (β = 1.001, *p* = 0.186) (Table 4). Ethnicity, marital status, socioeconomic status, and employment status were not associated with higher distress. Other demographic and clinical covariates are found in Table 4.

There was a significant difference between disease severity groups in terms of anxiety (F(2827) = 3.351, *p* = 0.036) (Table 4). Higher disease severity was associated with lower anxiety, between low and high disease severity groups only (*p* = 0.032). For depression, there was a significant difference between groups (F(2827) = 3.999, *p* = 0.019). Lower disease severity was associated with lower depression, between the low and medium-severity groups only (*p* = 0.020).

Mean anxiety and depression scores were both higher in AYAs with a pre-existing mental health condition, but differences were not statistically significant.

**Figure 1:**
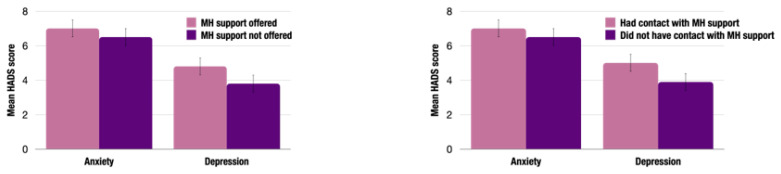
Relationship between distress scores and AYAs who were offered mental health support (**left**) and had contact with mental health support (**right**) at 6 months post-diagnosis.

AYAs with higher depressive symptoms were more likely to be offered (t(827) = −3.672, *p* < 0.001) and have contact (t(774)= 3.840, *p* < 0.001) with a mental health professional, as shown in Figure 1. There was no association between anxiety levels and being offered (t(827) = −1.617, *p* = 0.106) or having contact with (t(774) = −1.656, *p* = 0.098) a mental health professional.

**Figure 2:**
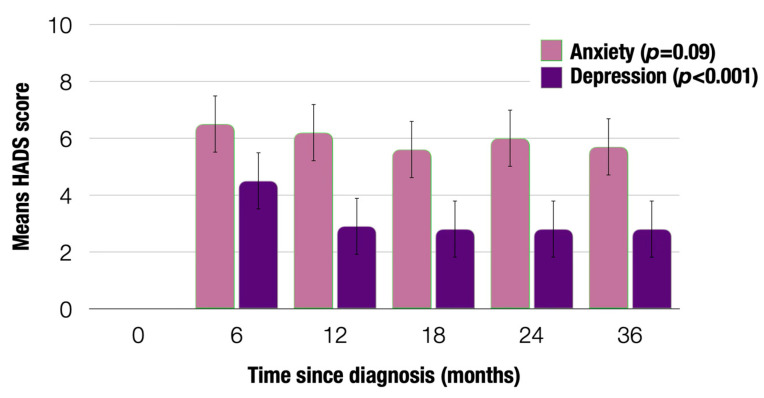
Change in distress across the five time points since diagnosis.

Across the five time points, anxiety did not change significantly (F(4109) = 2.069, *p* = 0.090) (Figure 2). However, depressive symptoms improved when comparing the first time point (6 months after diagnosis) to the subsequent four time points (12 to 36 months after diagnosis) (F(4109) = 10.460, *p* < 0.001). Of note, sample sizes across the time points were as follows: 6 months, n = 830; 12 months, n = 566; 18 months, n = 460; 24 months, n = 394; 36 months, n = 336.

### 3.3. Findings from the YAP Workshops

To help contextualize and interpret the study results, feedback was garnered through two participatory workshops with the BRIGHTLIGHT YAP in June 2024. Ten members (eight female and two male) were invited to the first online workshop, nine confirmed attendance and seven participated on the day. The second workshop was restricted to those who had attended the first, and all seven were present. Participants were all female, aged 14–20 at diagnosis, and had typical AYA cancers including sarcoma, lymphoma, and brain tumours. The group comprised a mix of patients who had recently finished treatment, those who had been off treatment for some time, and those now being treated for late complications of therapy.

**Figure 3:**
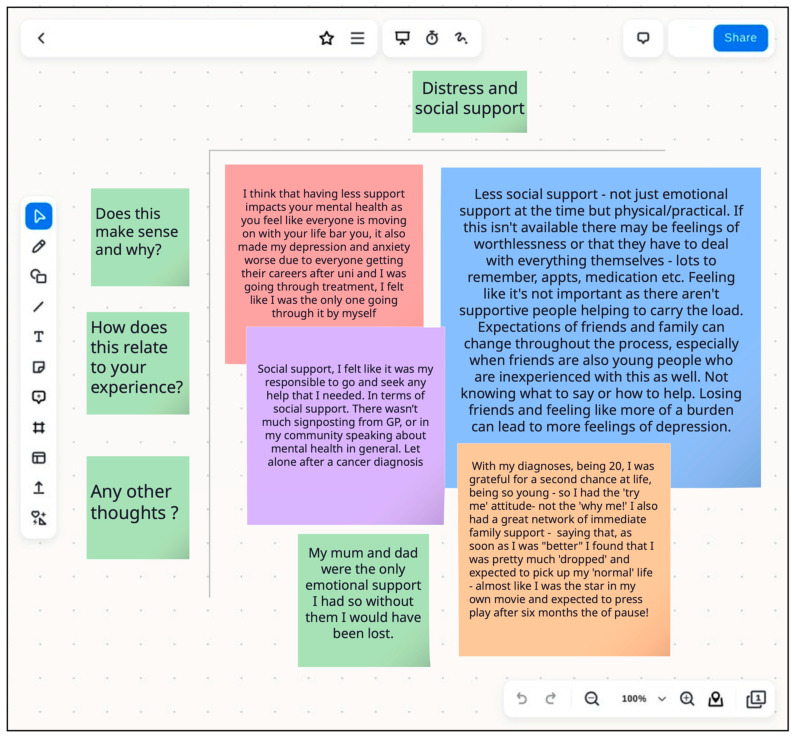
YAP whiteboard interpretation of the relationship between distress and social support based on their own experiences.

Overall, participants felt the topic of mental health in young people with cancer was important, not well acknowledged by their friends and family, and underexplored by their healthcare team during the first few years following diagnosis. Key study findings were explored in more detail with the YAP, including the above results from the primary and secondary research questions. Their interpretations are detailed below along with exemplar quotes. An example of a whiteboard with the variable ‘Social Support’ is included below (Figure 3).

In general, the YAP thought that higher levels of social support would be protective against distress, in contrast with the study findings. However, the discussion on this topic was extensive, and they identified both positive and negative impacts of social support on distress, summarized in Table 5.

An experience highlighted by multiple participants was a strong sense of duty to protect close family and friends from further emotional pain, by keeping their own feelings private. While most participants recognized they had several individuals they could rely on for emotional support, this recognition did not necessarily translate into them actively seeking out and sharing their feelings with those individuals:

“I’m actually really upset about this, [but] I had to be strong for everybody else. And I think that’s something that kind of gets missed a lot because you have a family member there. So my mom sat there crying, but she would cry the whole way home, and I had to make sure she was okay. So without, you know, going into too much negativity, I had to sort of be the strong one. And I think that definitely weighed on my mental health”.

Another common observation was that family and friends often anticipated an immediate return to normalcy following the end of treatment, when the young person was only beginning to process the emotions that come with cancer. One participant reflected that,

“…as soon as I was better I found that I was pretty much dropped and expected to pick up my normal, almost like I was the star in my own movie and expected to press play after 6 months of pause.”

The YAP thought the finding that female gender was associated with significantly higher anxiety and depression was not surprising. They reported that women may be more likely to talk about emotions than men, and that it may be more conventional for women to report distress and seek help:

“…I think it’s more socially acceptable to have to need to seek help for mental health and are, like, if I was having a bad day, I’d be a lot more vocal about it than my partner would be. And so I think it’s probably, personally, I think men try not to talk about things like that…”

They also wondered about emotional maturation occurring earlier in females, and about the differential impact of cancer treatment on female reproductive hormones, thereby influencing rates of distress.

The association of disease severity significantly predicting distress resonated with the YAP. Several reported a lack of awareness soon after diagnosis about the severity of their illness, for a variety of reasons. In some cases, information was communicated directly to parents or caregivers from the healthcare team, bypassing the young person. In others, incomplete or surface-level details may have been given to the young people:

“…it wasn’t till a few years later that I actually realized the severity [of the diagnosis].”

One participant wondered whether their young age and a retained sense of invincibility, at least initially, may have been protective for their mental health:

“I was very naïve and unaware of the severity of my cancer. I think this worked in my favour with my mental health.”

Others reported that the severity did not matter after a diagnosis was finally confirmed. The relief of having an explanation for their symptoms provided welcome validation. One participant thought that the focus on the physical aspects of treatment and recovery could distract from or protect against the mentally distressing aspects, while another felt conversely that physical limitations such as not being able to dance significantly worsened their mental health. It was reflected that each person’s situation and associated emotions were their own.

The YAP had several insights about the finding that there was no difference in distress between people with and without a pre-existing mental health condition. They thought that AYAs with a mental health diagnosis before cancer may already relate to appropriate support, which could position them to have better healthcare system navigation skills, feel more prepared to speak up about physical symptoms and be equipped with tools to cope with distress. Most thought that during the time of recruitment (2012–2014), there may have been under-reporting or under-detecting of mental health conditions, due to higher levels of stigma and barriers to accessing mental healthcare:

“I think there will be a lot of young people who just haven’t received a diagnosis yet. So then down the line, it may appear that if they are struggling mentally, it’s been, like, directly because of their cancer and what they’ve been through… But it could be that they may be already suffering with anxiety. But it was just kind of put down to being a teenager and, like, normal worries.”

Some participants wondered whether people might have already been imagining the ‘worst case scenario’, and thus were not taken aback when the diagnosis eventually came:

“If you’re a person who tends to worry a lot, when the thing you’re worrying about actually happens, you suddenly become very calm. Because that disaster I’ve been waiting for is here.”

The YAP shared their own experiences of distress over time from diagnosis (responses summarized in Table 6). Most reported higher overall levels of anxiety than depression, in line with the study results. Many reported a decrease in overall distress across the time points, corroborating findings that depression was significantly lower after the 1-year mark.

Several participants wished they had been asked about their mental health as they progressed through treatment and in the months and years afterward but they were not given the opportunity. The YAP noted that while there were many common emotional reactions over time, each person’s experience and feelings were personal and unique.

## 4. Discussion

This study contributes novel insights into the mental health of young people with cancer by providing a deeper understanding of risk and protective factors related to psychological distress in a large, longitudinal sample of AYAs. Six months after diagnosis, young people in our cohort reported anxiety and depression rates (borderline plus moderate/severe) similar to other studies of the AYA population, with anxiety ranging from 8 to 55% and depression from 13 to 47% [31,38,39,40]. Anxiety levels decreased with age, and females reported higher rates of distress, anxiety, and depression, aligning with results from several studies [46,47,48]. The YAP agreed that females might find it more socially acceptable to express emotions and seek help.

The first aim of this study was to investigate the relationships between distress and social support, disease severity, pre-existing mental health conditions, and contact with mental health professionals during treatment. Contrary to the proposed hypotheses, increased distress was associated with higher levels of social support and less severe illness. Existing research suggests the presence of close family and friends generally mitigates distress [76,77,78]. The YAP noted both positive and negative impacts of social support, including the benefits of practical help versus the burden of suppressing their feelings to protect loved ones, which are concepts supported by the literature [76,79]. It is also possible that current quantitative measures of social support do not capture the complexity and nuance involved in social interactions young people experience after a cancer diagnosis. A final possibility is that distress itself may have evoked greater social support.

Another unexpected finding was that lower disease severity was associated with higher anxiety scores at the first time point, which persisted after controlling for covariates. There is a dearth of research on this topic in AYAs [80], but the YAP suggested this could be due to an initial lack of awareness about the severity or the invincibility of youth. Another possibility is that young people did not allow the severity to define them, i.e., other illness-independent factors were driving their emotional response [81,82].

Additionally, there was no significant relationship between the presence of a pre-existing mental health condition, a screen-detectable target for early psychological intervention, and distress, likely due to small numbers of AYAs disclosing a pre-existing condition. Response bias should also be noted, as these were self-reported responses to an interviewer-administered questionnaire. Although little evidence exists on this issue, the YAP posited that AYAs with mental health diagnoses before cancer might benefit from existing healthcare connections and coping tools, helping manage distress. They also commented that higher levels of stigma and barriers to accessing mental healthcare during recruitment more than 10 years ago may have led to under-identifying these conditions.

Finally, anxiety levels did not correlate with being offered or having contact with support, but AYAs with higher depression scores were more likely to be referred to and connected with a mental health professional. In this study, distress scores were not reported back to the treating clinicians, indicating the possibility that either clinicians were good at detecting distress in other ways, or patients (and/or caregivers) were good at advocating for their mental health needs, or both. In a large cohort study comparing rates of mental health service utilization between 639 AYAs with cancer and 29,793 without, those with cancer were more likely to undergo psychotherapy [83]. A qualitative study in AYAs with cancer highlighted that offering mental health services to all patients at multiple time points enhanced engagement with and utilization of those services [84].

The second aim of the study was to explore the change in distress along the cancer trajectory, predicting a decrease. This was borne out for depression, which decreased significantly after the first time point, but not for anxiety, which remained stable. Due to a lack of longitudinally designed research on distress in AYAs with cancer, changes in mental health symptoms over time are poorly understood. There are a small number of long-term follow-up studies, each varying in duration, research design, measures of distress, AYAs age range, and cancer type [44,49,58,83]. Evidence from these studies indicates mixed outcomes, with rates increasing, decreasing, or remaining stable over time.

The YAP described highly personalized distress trajectories depending on life stage, specific diagnosis and treatment, and amount of educational or vocational support. Anxiety was the more dominant emotional state over depression, with both declining over time. Nonetheless, the YAP emphasized the need for emotional support beyond the end of the study period, because, despite a general decrease, symptoms did not fully dissipate.

This study encountered common cohort study limitations, such as selection bias, differential loss to follow-up, and missing data [85,86]. Treatment status (on/off treatment) was also not analyzed, and the sample’s predominantly white ethnicity from England narrows the generalizability of the findings. Moreover, given that the cohort’s data are over a decade old, it represents a historical perspective of medical and mental healthcare, which has undoubtedly evolved [87]. The analyses in this study were confined to the outcome measures captured in the survey, precluding the examination of additional mental health variables and confounders, such as specific diagnoses, or more granular surveys on depression, anxiety, grief, post-traumatic stress, and suicidal ideation. Moreover, the database lacked insights into distress, including narrative accounts of symptomatology at various time points or the effects of mental health interventions. Although few measures have been psychometrically tested within this demographic, often being validated in populations of younger children or older adults, the HADS and MSPSS have been consistently utilized in AYAs cancer research, affirming their effectiveness in assessing the intended constructs [68,88,89].

While this study benefits from a longitudinal design, certain analyses were conducted cross-sectionally (e.g., at 6 months after diagnosis) to provide a detailed understanding of distress and its correlates early in the cancer trajectory. Cross-sectional analyses are particularly valuable in early survivorship, where timely identification of psychosocial risk factors can inform real-time clinical decision-making and targeted interventions. By capturing distress levels at distinct time points, this approach allows for immediate assessment of mental health needs, ensuring that support can be mobilized when most needed.

However, because these analyses were conducted early in the disease course, the positive association between distress and social support may reflect the process of seeking and utilizing support in response to distress, rather than social support acting as a prospective protective factor. Examining these relationships prospectively within the available longitudinal dataset would provide a clearer picture of how social support influences distress over time. Additionally, while cross-sectional analyses allow for efficient subgroup comparisons, they do not capture individual trajectories of distress or establish causality. As distress in AYAs is dynamic, future research could incorporate time-dependent models, such as linear mixed-effects models or growth curve analysis, to better understand long-term psychosocial trends.

Finally, although the YAP’s input was vital to the research, their involvement had certain constraints. Notably, due to the absence of male participants and being self-selected and well-versed in cancer research and PPI, the group might not necessarily reflect the broader views of all AYAs [62].

Despite these limitations, the large, longitudinal dataset used in this study allowed for a broad exploration of the mental health characteristics of young people and how distress changed over time. As described in more detail by Taylor and colleagues [90], engaging young people in research from the very beginning and implementing a centralized system for tracking patients seem to be crucial strategies for enhancing recruitment and retention.

Furthermore, the YAP workshops provided crucial interpretations of study findings, filling existing literature gaps with insightful and complex hypotheses. The YAP validated the importance of the topic and the need for continued research in this area. Contrary to prevailing descriptions of this population as vulnerable [91,92], which often leads to their exclusion from research participation [62], the YAP freely shared their feedback, particularly eager that their input would be used to inform future interventions.

Future research on the topic of mental health in AYAs with cancer should include longer follow-up periods and larger sample sizes. Sensitivity analyses examining the differences between distress levels in those who participated across all time points versus those lost to follow-up would enhance the validity of the results. Additional prospective analysis of the longitudinal data could help offer a clearer picture of the impacts of variables such as social support on distress over time. With respect to social support in particular, the surprising finding that higher levels were not protective against distress is worthy of further study. A clearer understanding of that relationship could help minimize harm and harness the benefits of social support for AYAs with cancer.

Prospective qualitative exploration of distress along the disease trajectory should be undertaken, to understand patterns of distress over time and experiences of accessing professional mental healthcare during or after treatment. Synthesizing quantitative and qualitative data would provide a more detailed picture of the psychological burden in this population, helping inform the design and evaluation of individualized screening and intervention tools. While reported rates of pre-existing mental health conditions in this study were low, between suspected underreporting at the time of the study and increasing rates of mental health conditions over time since the study was conducted, current rates are likely to be much higher. Newer studies examining these rates using multiple methods could help strengthen the recommendation that screening should be routinely offered to facilitate early detection and provide proactive interventions.

Clinical care improvements could include facilitating the early detection and treatment of mental health concerns shortly after diagnosis, addressing modifiable risk factors and bolstering protective factors. Findings also underscore the importance of sustained psychosocial support during and after treatment, and enlightening healthcare professionals about developmental explanations for distress in AYAs at different stages of their cancer experience [48]. An open dialogue about mental health in hospitals can not only facilitate better referral pathways but also reveal service shortfalls, thereby informing advocacy initiatives aimed at filling these gaps.

In a recent priority setting exercise, AYAs themselves identified the top AYAs research priority as finding a “psychological support package that improves psychological well-being, social functioning and mental health during and after treatment” [93]. Young people should continue to be included as active co-designers in all elements of research, as they have been in the BRIGHTLIGHT study. Given appropriate scaffolding from researchers, young people can contribute immensely to healthcare research. The advantages to AYAs, the research team, and the broader AYA community far outweigh any obstacles [62].

## 5. Conclusions

Several key conclusions may be drawn from this study. First, while this large cohort has similar rates of overall distress to other AYA studies, high levels of social support and low disease severity were identified unexpectedly as potential risk factors for distress. Identifying the presence of a support network alone may not capture the distress young people feel about their cancer’s impacts on that network and thus may not necessarily equate to active confiding or soliciting support. Guidance on improving emotional communication can help young people and their support networks navigate the feelings associated with a cancer diagnosis. Furthermore, severe disease does not necessarily register with a young person as emotionally detrimental. These points reinforce the importance of avoiding assumptions about factors affecting a young person’s emotional state. Second, pre-existing mental health conditions are important targets for screening initiatives, particularly due to increasing rates of mental health conditions in young people. Third, while professional mental health support is being offered to AYAs with cancer, rates of referral could be higher. Fourth, young people need emotional support long after their diagnosis, given the persistent nature of symptoms over time. Finally, participation and engagement from young people themselves in research and clinical programming is vital and rewarding for all. Insights from this study will help shape the creation of developmentally tailored screening approaches and intervention strategies, to alleviate distress and improve the overall well-being of this unique and understudied population.

## Figures and Tables

**Table 1:** Sociodemographic characteristics of the BRIGHTLIGHT cohort at the first time point (6 months after diagnosis).

Characteristic		Frequency	% ^a^
Age at diagnosis (years)			
	Mean (SD)	20.1 (3.3)	
Gender			
	Male	457	55.1
	Female	373	44.9
Ethnicity			
	White	730	88.0
	Mixed	14	1.7
	Asian	61	7.3
	Black	15	1.8
	Chinese	4	0.5
	Other	6	0.7
Marital status			
	Married/civil partnership	26	3.1
	Cohabiting	93	11.2
	Single/divorced	606	73.0
	Missing data	105	12.7
Geographic location			
	North East England	44	5.3
	NW	106	12.8
	Yorkshire	100	12.0
	East Midlands	107	12.9
	West Midlands	120	14.5
	London	165	19.9
	South East England	98	11.8
	Sound West England	90	10.8
Socioeconomic status (IMD quintile)			
	1—most deprived	184	22.2
	2	136	16.4
	3	156	18.8
	4	182	21.9
	5—least deprived	152	18.3
	Missing data	20	2.4
Employment status			
	Working full/part time	257	31.0
	Education	274	33.0
	Other work (apprentice, intern, volunteer)	17	2.0
	Unemployed	31	3.7
	Long term sick leave	126	15.2
	Not seeking work	125	15.1

Note. SD, standard deviation; IMD, Index of multiple deprivation. ^a^ Column percentages displayed.

**Table 2:** Clinical characteristics of the BRIGHTLIGHT cohort at 6 months after diagnosis.

Characteristic		Frequency	% ^a^
Cancer type—Birch classification [75]			
	Leukemia	106	12.8
	Lymphoma	267	32.2
	Central nervous system	33	4.0
	Bone	70	8.4
	Sarcoma	59	7.1
	Germ cell	154	18.6
	Skin	31	3.7
	Carcinoma (not skin)	100	12.0
	Other ^b^	10	1.2
Time to diagnosis (days) ^c^			
	Mean (SD)	125.3 (172.6)	
	Median (IQR)	62.0 (29.0, 153.0)	
Disease severity at diagnosis			
	Least severe	461	55.5
	Intermediate	194	23.4
	Most severe	175	21.1
Prognostic score			
	<50%	127	15.3
	50–80%	239	28.8
	>80%	460	55.4
	Missing data	4	0.5
Treatment type ^d^			
	SACT only	271	32.6
	RT only	14	1.7
	Surgery only	117	14.1
	SACT and RT	106	12.8
	SACT and Surgery	181	21.8
	SACT, RT, and Surgery	77	9.3
	RT and Surgery	36	4.3
	Transplant	19	2.3
	Other ^e^	9	1.1

Note. SACT, systemic anticancer therapy; RT, radiation therapy; ^a^ Column percentages displayed. ^b^ Other diagnoses included Birch grouping categories 9–12 (see Birch et al., 2008 [75] for more detail). ^c^ From first symptom to diagnosis. ^d^ In the first 12 months since diagnosis. ^e^ Other was recorded if patients were in a watch and wait category at diagnosis and then received SACT, RT, and/or surgery. IQR, interquartile range.

**Table 3:** Psychosocial characteristics of the BRIGHTLIGHT cohort 6 months after diagnosis.

Characteristic			Value
HADS ^a^			
	Anxiety score, mean (SD)		6.89 (4.39)
		Borderline, n (%)	160 (19)
		Moderate/severe, n (%)	172 (21)
	Depression score, mean (SD)		4.62 (3.68)
		Borderline, n (%)	120 (15)
		Moderate/severe, n (%)	55 (7)
MSPSS, median (IQR)			
	Support—friends		1.75 (1–2.75)
	Support—family		1.25 (1–2)
	Support—significant others		1 (1–2)
	Total support		1.5 (1.1–2.2)

Note. HADS, Hospital Anxiety and Depression Scale; MSPSS, Multi-dimensional Scale of Perceived Social Support. ^a^ Borderline = 8–10, moderate/severe > 11.

**Table 4:** Associations between distress symptoms (anxiety and depression) and factors known to impact mental health at 6 months after diagnosis ^a^.

Distress Symptom			Test Statistic	*p*-Value	95% CI
Anxiety					
	Age		β = 1.022	0.002	0.008 to 0.035
	Gender		β = 0.696	<0.001	−0.420 to −0.305
	Social support				
		From family	β = 1.028	<0.001	1.696 to 1.799
		From friends	β = 1.030	<0.001	1.631 to 1.732
		From significant others	β = 1.009	0.003	1.818 to 1.915
		Total	β = 1.148	<0.001	0.108 to 0.168
	Disease severity				
		Between groups	F(2827) = 3.351	0.036	0.000 to 0.023
		Low vs. Medium		1.000	−0.774 to 1.024
		Low vs. High		0.032	0.0610 to 1.927
		Medium vs. High		0.172	−0.226 to 1.964
	Pre-existing mental health condition		U = 385.500	0.162	−1.241 to 0.276
	Offered contact with a mental health professional		t(827) = −1.617	0.106	−0.287 to 0.028
	Had contact with a mental health professional		t(774) = −1.656	0.098	−0.280 to 0.024
Depression					
	Age		β = 1.001	0.186	−0.005 to 0.027
	Gender		β = 0.729	<0.001	−0.388 to −0.246
	Social support				
		From family	β = 1.033	<0.001	1.254 to 1.378
		From friends	β = 1.041	<0.001	1.119 to 1.242
		From significant others	β = 1.015	<0.001	1.367 to 1.485
		Total	β = 1.192	<0.001	0.140 to 0.212
	Disease severity				
		Between groups	F(2827) = 3.999	0.019	0.000 to 0.025
		Low vs. Medium		0.020	−1.610 to −0.100
		Low vs. High		0.390	−1.270 to 0.290
		Medium vs. High		1.000	−0.560 to 1.280
	Pre-existing mental health condition		U = 431.000	0.301	−1.167 to 0.349
	Offered contact with a mental health professional		t(827) = −3.672	<0.001	−0.452 to −0.136
	Had contact with a mental health professional		t(774) = 3.840	<0.001	−0.450 to −0.145

Note. CI, confidence interval. ^a^ Univariate analyses.

**Table 5:** Impacts of social support from family and friends on mental health.

Source of Support	Positive Impacts	Negative Impacts
Family	‘Managing the load’: Practical support with scheduling and taking to appointments, taking medication	‘Protecting family’, having to manage family emotions and being the strong one Having support early on from new family members appearing and then ‘dropped’ when ‘better’ Lack of available support for family members experiencing distress Feeling overwhelmed by ‘too many people around’, feeling pressure to see them when not feeling well enough Loss of identity if forced temporarily to give up a caregiving role
Friends	Coming to visit when in hospital or at home, when too sick to go out	Seeing friends move on with their lives but feeling held back due to impact of treatment on activities Losing contact with friends Feeling like a burden on friends Expectations from friends to ‘be like before’, hard for friends to understand post-treatment symptoms like fatigue, pain Friends may not want to discuss difficult feelings or think about ‘worst case scenarios which you as the patient may be having to face’ Having lots of friends around but not wanting to acknowledge what’s happening could ‘feel more isolating than not having them at all’

**Table 6:** Perspectives of the YAP on patterns of distress over time from diagnosis.

**Time**	**Anxiety (Things I Was Worrying About)**	**Depression (Things I Was Sad About)**
6 months	Losing friends, becoming disconnected Treatment (surgery) and its implications Side effects of chemotherapy: how bad each cycle would feel physically Fear of recurrence Returning to school/work	Feeling left behind socially, academically/workwise Coping with permanent physical changes Grieving lost identities, friendships
12 months	Feeling “normal” again Adapting to new limitations Returning to school or work Needing more treatment, associated uncertainty Scans	Coming to terms with new limitations Difficulties with ongoing treatment, thinking about stopping
18 months	Needing school/work accommodations “Normal teenage worries” (e.g., sitting GCSEs) ‘Survivor guilt’	Struggling with being well, but ‘nowhere near what it was before’
2 years	Adjusting back at school/work Fear of recurrence Starting a ‘new life’ Survivor guilt Late effects starting Adapting to new limitations	No depression symptoms
3 years	Worries waning, ‘I can still live a good life’ Late effects progressing Difficulties securing work due to lack of accrued experience	No depression symptoms

Note. GCSEs, General Certificate of Secondary Education.

## Data Availability

Further details of the BRIGHTLIGHT programme of work is available through the study website (www.brightlightstudy.com, accessed on 30 January 2025). We welcome collaboration, for general data sharing enquiries please contact R.M.T. (rtaylor13@nhs.net).
